# Tick paralysis induced by *Ixodes gibbosus*: enigmatic cases in domestic mammals from Cyprus

**DOI:** 10.3389/fvets.2024.1416501

**Published:** 2024-06-06

**Authors:** Anastasia Diakou, Angelique Foucault-Simonin, Giannakis Antoniou, Alejandro Cabezas-Cruz, Gábor Földvári

**Affiliations:** ^1^Laboratory of Parasitology and Parasitic Diseases, School of Veterinary Medicine, Aristotle University of Thessaloniki, Thessaloniki, Greece; ^2^Diagnostics and Laboratory Research Task Force, Balkan Association for Vector-Borne Diseases, Novi Sad, Serbia; ^3^ANSES, INRAE, Ecole Nationale Vétérinaire d’Alfort, UMR BIPAR, Laboratoire de Santé Animale, Maisons-Alfort, France; ^4^Independent Practitioner, Polis Chrysochous, Cyprus; ^5^Institute of Evolution, HUN-REN Centre for Ecological Research, Budapest, Hungary; ^6^Centre for Eco-Epidemiology, National Laboratory for Health Security, Budapest, Hungary

**Keywords:** *Ixodes gibbosus*, tick paralysis, Cyprus, free ranging, sheep, goat, dog, cat

## Abstract

Tick paralysis is a potentially fatal condition caused by toxins produced and secreted by tick salivary glands. This survey presents clinical and epidemiological observations of tick paralysis cases in domestic animals in Cyprus. Local veterinarians report typical tick paralysis cases occurring in goats, sheep, dogs, and cats. The animals suffering from paralysis are free from other neurological diseases, have blood and biochemical parameters within normal ranges, and recover fast by simply removing the ticks found predominantly on the head and around the neck. Tick paralysis cases occur in a specific geographic area of Cyprus (Akamas peninsula), from September through March, but not every year. Instead, the phenomenon has 2 periodic cycles of occurrence, a 3- and a 7-year cycle. The 2 cycles are differentiated by severity based on the number of affected animals and the resulting losses. As described for other tick-borne diseases, these cyclic patterns may be attributed to external factors, self-oscillations of the disease system, or the combined action of these mechanisms. Ticks collected from a recent paralysis case in a goat were morphologically and molecularly identified as *Ixodes gibbosus*. Efforts should be made to characterize the specific toxins involved in tick paralysis and to develop a vaccine, which could prevent significant losses of small ruminants, especially in free-ranging farming systems, a prevalent management approach observed in Cyprus and various regions worldwide.

## 1 Introduction

Tick paralysis is caused by toxins, that reduce neurotransmission in hosts, produced and secreted by tick salivary glands (1). This condition develops as a flaccid, ascending paralysis that, if left untreated, can lead to death. Simply removing the responsible ticks, results in complete recovery of the affected animal or human. The most potent paralysis-inducing tick known to date is *Ixodes holocyclus*, found in Australia. The responsible neurotoxins of this tick (holocyclotoxins) have been used to produce commercially available tick antiserum (2). However, there are at least 73 tick species associated with tick paralysis cases in different animal species and humans, worldwide (1, 3). Ticks synthesize and secrete toxins responsible for tick paralysis in a gradual manner, with heightened secretion occurring during the later stages of feeding. As a result, clinical signs usually develop several (4–8 days) after tick attachment (3). Consequently, tick paralysis is associated with hard ticks (Ixodidae) at all developmental stages (but not males) and with larvae of soft ticks (Argasidae), due to their prolonged attachment to the host (3). In experimentally induced tick paralysis, the secretion of neurotoxins is limited to ixodid females and coincides with a definite repletion phase (4).

Toxins must exceed a certain concentration, in mg per ml of host blood, for paralysis to manifest (1). Therefore, the tick developmental stage, the tick species and the size of the host are critical factors. As a result, the number of ticks required to cause paralysis varies significantly (5), i.e., from hundreds of nymphs to a single female (3). The evolutionary and adaptive role of paralytic toxins produced by ticks is not yet fully understood, however, hindering grooming to prevent tick removal has been suggested (6). In most cases, information about the molecular profile of tick toxins is lacking (5), although ticks and their salivary proteins possess similarities attributed to venomous animals (7).

Most cases of tick paralysis in animals and humans are described in Australia, the United States, and Canada, while they are rarely reported in Europe (1, 8). Tick paralysis is not a reportable condition and thus probably it is more common and widespread than currently known but remains under-reported and underestimated. In animals, it is often diagnosed and reported when outbreaks occur (9, 10). Building upon this foundation and with the aim of offering fresh insights into tick paralysis, the present survey describes clinical and epidemiological observations on tick paralysis cases in domestic animals in Cyprus, characterized by some unique traits, i.e., the specific pattern of periodicity and the restricted geographical area of occurrence, that grant an enigmatic nature to this phenomenon.

## 2 Methods and results

### 2.1 Epidemiological data

**Figure 1:**
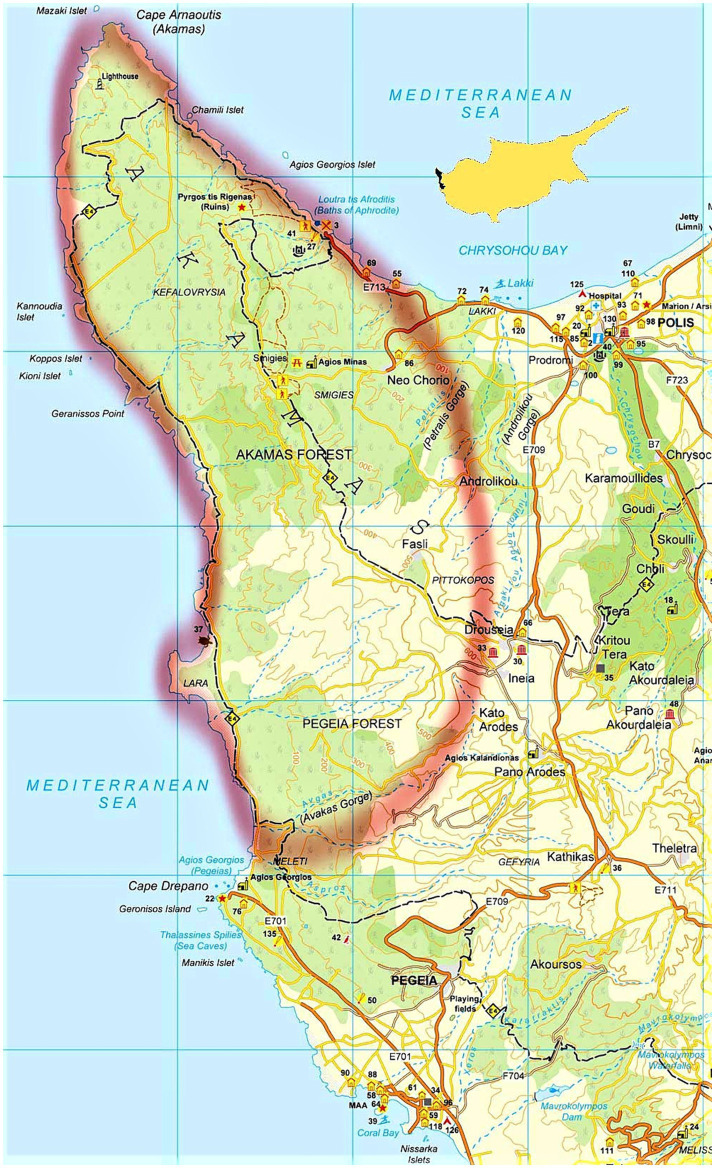
The Akamas peninsula, designated with the red line, where tick paralysis cases in animals are reported in Cyprus.

Tick paralysis cases are reported from a specific geographic area of Cyprus, a peninsula at the northwest extremity of the island called Akamas (Figure 1). Akamas is a region in the eastern Mediterranean with unique geomorphology, microclimate, habitat, and diverse flora and fauna. A significant number of endemic or rare plant species can be found in Akamas, making it a valuable area for conservation. In fact, Akamas has been designated as a National Park to protect its well-preserved ecosystem (11). There are two rearing systems for small ruminants in this area: a semi-intensive and a free-ranging system. In the latter, the animals are allowed to roam freely, graze on natural vegetation, and are only caught by their owners once or twice a year for health checks, shearing, and/or to collect milk or meat.

Local veterinarians and farmers report tick paralysis cases occurring in semi-intensively reared but mostly in free-ranging small ruminants, and sporadically in dogs and cats. The cases are recorded between September and March. Strikingly, the cases display two distinguished periodic cycles, i.e., a 3-year and a 7-year cycle, differentiated by severity based on the number of affected animals and the resulting losses, with the 7-year occurrence being more severe. In fact, the local farmers have a common name for the condition, known for hundreds of years. In Greek, the name is “Laokourtouno to Trichronitiko” for the 3-year cycle, and “Laokourtouno to Eptachronitiko” for the 7-year cycle. The first word of the name refers to the tick causing paralysis, while the second word refers to the periodic cycle of the phenomenon (“trichronitiko” and “eptachronitiko” mean occurring every 3, and every 7 years, respectively).

In an effort to gather more information about the epidemiology of tick paralysis cases in Akamas, a questionnaire (Supplementary file) was prepared and distributed to 22 local sheep/goat owners. Of the 22 flocks, 8 were free-ranging (4 goat and 4 mixed sheep and goat flocks) and 14 were reared in a semi-intensive system (1 goat, 7 sheep and 6 mixed sheep and goat flocks). According to the information collected, tick paralysis is a well-known phenomenon in the area, as all 22 farmers questioned have repeatedly observed tick paralysis in their animals. Young and free-ranging animals are especially vulnerable to this condition, which tends to occur primarily during the autumn and winter months. Farmers reported through the questionnaire that they observe a similar number of deaths in both sheep and goats, and they have also witnessed two cases in dogs and one in a cat. The animal losses, recorded at the 7-year occurrence often exceed a count of 50 per free-ranging flock (the number of deaths reported by the farmers in the questionnaire range from 5 to over 50), particularly affecting younglings. It is worth noting that many free-ranging animals developing tick paralysis are never retrieved by their owners, so the true extent of the losses may be underestimated. To prevent the condition, acaricides are commonly administered, usually during the years when the problem is expected to occur. In some cases, farmers keep the animals restricted in the farm to avoid tick infestation.

### 2.2 Clinical observations in tick paralysis cases occurring in Cyprus

The animals affected by the tick toxin are found with flaccid ascending paralysis starting from the hind extremities, expanding to the anterior part of the body, and occasionally accompanied by pedaling. Progressively, the animals lose the swallowing reflex, and show an inability to feed, dyspnoea, and pulmonary oedema (likely due to heart failure). Uraemia, due to urinary retention and abortions have also been recorded in some affected animals. The animals eventually die if the ticks remain attached. Ticks are found in different parts of the body but mainly on the head and around the neck. The affected animals are free of pre-existing clinical conditions, have blood/biochemical workout results within normal ranges, and a normal temperature. Differential diagnosis excludes conditions with a similar clinical picture, including vitamin B1 deficiency, hypocalcaemia, coenurosis, listeriosis, and pregnancy toxaemia (12).

According to observations, the number of ticks required for paralysis to manifest is 5–6 in lambs and goat kids, and 18–32 in adult sheep and goats. The onset of paralysis is observed when already engorged females can be spotted on the affected animals, mostly on the head and around the neck. The outcome in different cases depends on the stage of paralysis in which the animal is found. In fact, these cases show similarities in regard to the progression of symptoms and duration of recovery with those reported in North America (fast recovery after tick removal), rather than in Australia (progression of paralysis for 24 to 48 h after tick removal) (1). Farmed animals are usually found in an early stage by the farmers and the removal of ticks (mechanical or/and with the use of acaricides) results in total recovery between 4 and 24 h. Free-ranging animals, however, are often found in a progressed stage of paralysis. In this case, the recovery may take longer (up to 48 h), or in some cases, the animals die, mainly because of respiratory failure.

### 2.3 Laboratory investigations in a recent case

**Figure 2:**
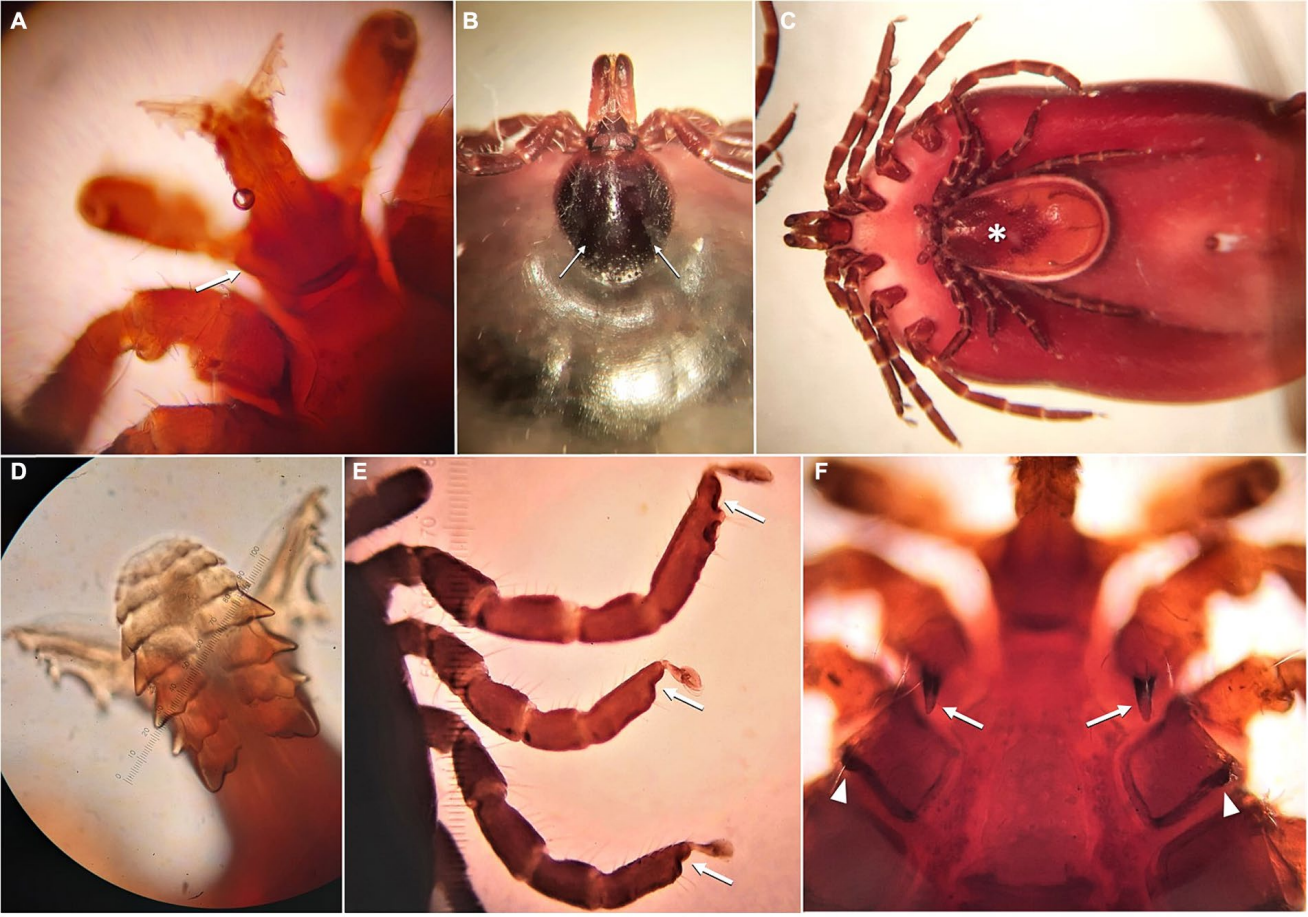
*Ixodes gibbosus* ticks identified in a tick paralysis case in Akamas, in Cyprus. **(A)** Male, basis capituli with indistinct auriculae (arrow); **(B)** Female, scutum with scapular grooves (arrows); **(C)** Female and male (asterisk) in copulation; **(D)** Hypostome of male with seven rows of serrated ridges; **(E)** Tarsi, bluntly stepped toward the claws (arrows); **(F)** Long internal spurs of coxae 1 (arrows) and indistinct external spurs on coxae 2 (arrowheads) to 4 (not shown).

Ticks collected from a recent tick paralysis case in a goat kid from Akamas peninsula in Cyprus were morphologically identified as *Ixodes gibbosus* (Figure 2) (13–15).

The morphological identification was confirmed by molecular methods. Briefly, tick DNA was extracted using the Nucleospin Marcherey Nagel kit (Macherey-Nagel, Hœrdt, France). The primers for the genus *Ixodes*, 16S-F 5’-TTAAATTGCTGTRGTATT-3′ and 16S-R1 5’-CCGGTCTGAACTCASAWC-3′, were used and the product (455 bp) was sequenced revealing 99% similarity with GenBank accession number AF549846.1, and 98.6% with accession numbers MT302763.1 and MT302762.1. The primers for the cytochrome oxidase subunit I (*cox1*) gene, HCO2198 5′-TAA ACT TCA GGG TGA CCA AAA AAT CA-3′ and LCOI1490 5′-GGT CAA CAA ATC ATA AAG ATA TTG G-3′ were used and the product (710 bp) was sequenced revealing a 99% similarity with GenBank accession number MT308591.1, and 99% with accession number MT308590.1. Primers HCO2064 5′-GGT GGG CTC ATA CAA TAA ATC C-3′ and 5’-GCC ATT TTA CCG CGA TGA-3′ were also used, and the product (860 bp) was sequenced revealing a 99% similarity to GenBank accession number MT308591.1, and 98% with accession number MT308590.1. All accession numbers were generated from *I. gibbosus* specimens found in Turkey.

## 3 Discussion

The reported cases of tick paralysis in the Akamas peninsula in Cyprus expand our limited knowledge of this enigmatic disease in Europe and of the role of *I. gibbosus*, a neglected European tick species (15). The observations reported herein describe two unique characteristics regarding tick paralysis epidemiology: (a) the well-defined, restricted area of occurrence (i.e., the Akamas peninsula), and (b) the firm periodicity of cases. To the best of the authors’ knowledge, these characteristics have not been previously described in this context. Furthermore, this is the first time that ticks collected from an animal suffering of tick paralysis in Cyprus, have been identified (*I. gibbosus*). The tick fauna of Cyprus compromises of at least 11 different species (16), thus it cannot be ruled-out that other tick species may also be involved in the paralysis cases recorded in Akamas peninsula. Concerns about tick paralysis are warranted due to the significant number of animals affected in each “outbreak,” leading to important economic consequences.

To date, *I. gibbosus* has not been extensively studied (15). *Ixodes gibbosus* is an exophilic, three-host tick with a winter activity distributed in areas considered too warm and dry for *Ixodes ricinus* and replaces the latter in several Mediterranean countries (15). In Greece, *I. gibbosus* is mainly active between September and April, in Israel between November and February, and in Cyprus between November and April (13, 16). To date, there have been no systematic studies about the vectorial role of *I. gibbosus* in tick-borne pathogen transmission (15). However, *I. gibbosus* has been identified before as the cause of sporadic or epizootic tick paralysis (13). In fact, Saratsiotis (13) reported epizootic tick paralysis by *I. gibbosus* at the end of November 1965 in an area of Central Greece. The author attributes the phenomenon to the climatic conditions of that year, i.e., delayed rainfalls with relatively high temperatures and prolonged drought that had persisted late into the season. Specifically, it was assumed that these conditions led to a considerable immobilization of ticks in their shelters, awaiting favorable microclimatic conditions for active host searching. In parallel, the high temperatures in that year accelerated the life cycle of a certain number of ticks, which, under usual conditions, would not have reached the adult stage until the following spring. Therefore, a massive emergence of ticks ensued as soon as the late rains occurred. This epizootic lasted almost a month and tick paralysis cases disappeared with the use of acaricides. The arrival of the first cold weather also contributed to the ceasing of massive infestations (13).

In Cyprus, *I. gibbosus* is associated with vegetated areas (16) and it is not found in dry environments (17). In any case, *I. gibbosus* is a common species on the island (15, 16, 18). To explore the herein described geographic restriction of tick paralysis in Akamas, it would be interesting to record the tick fauna composition and the *I. gibbosus* population dynamics in this area. Although it is not easy to speculate about the geographic limitation of the phenomenon, differentiated populations of some tick species are likely to harbor different microbiomes or symbionts that may play a role in the synthesis of proteins acting as neurotoxins (19). Hypothetically, the microbiome of *I. gibbosus* propagated by the local micro-environment (endemic flora and fauna) and habitat, may form the conditions for toxin synthesis. This scenario is supported by the fact that most bacterial endosymbionts are present in tick salivary glands (20).

The periodic 3- and 7-year occurrence of tick paralysis in Akamas is also a phenomenon worth studying. Thus far, there is no evident reason or any identifiable factor that explain the periodic cycle of tick paralysis “outbreaks” or epizootics in Cyprus. For example, no agricultural burning of fields or any other human activity that could be linked to this phenomenon has been observed. Similarly, no climatic condition has a regular multiannual, periodic manifestation. Cyclic occurrence patterns previously described for tick-borne diseases, e.g., tick-borne encephalitis, have been attributed to external factors or to self-oscillations of the disease system, or a combination of these and other similar mechanisms, but these periodic cycles are inter-annual (21, 22). Additionally, predictions for tick population densities based on climatic conditions can only be attempted for the next tick period or, at the most, for a short forecasting model (21, 23). To the best of the authors’ knowledge, a long periodic cycle of several years, as reported in the present survey, has never been described in tick-borne disease. One possible explanation is that the phenomenon results from the matching culmination of various factors having different cycle lengths (22). For instance, there may be factors that synchronize tick population oscillations, linked to temperature, humidity, microhabitat, and different developmental stages of ticks and life cycle duration, which can be between 2 to 6 years (24, 25). Data on beech tree seed production (masting) from the literature indicate that the abundance of *I. ricinus* nymphs can increase dramatically 2 years after a masting event (26). Similar periodic vegetational changes might shape *I. gibbosus* population fluctuations. Additionally, rodent population cycles and periodical outbreaks (27) in the area, could be a factor influencing tick populations, through a periodic abundance of hosts for tick larvae and nymphs. Furthermore, host migration in different pastures and environments — as is the case for the free-ranging animals in Akamas — may influence tick population dynamics (28). In fact, local farmers report higher tick population density during the years of tick paralysis “outbreaks,” an assertion that needs to be studied and confirmed. To serve this end, it would be useful to investigate any fluctuation in tick population dynamics (species and abundance) during the period between the tick paralysis occurrences.

Taking into account all the above, the enigmatic tick paralysis epizooties in Cyprus merit further investigation. Also, considering that *I. gibbosus* has been found feeding on humans (13), enrichment of our knowledge about paralysis caused by this neglected tick species is essential to address public health concerns. In accordance with the DAMA protocol, an acronym referring to the actions: Document, Assess, Monitor, and Act (29), it would be important to monitor tick paralysis occurrence in domestic animals and humans, and to document any differences in tick-species or population-density fluctuations in the course of the 3- and 7-year periodic cycles. Finally, a key-advancement toward a solution of the problem would be the characterization of the specific toxins involved in these paralysis cases, as this could lead to the development of a vaccine, at least in a small-scale, in-house production (30). Such a vaccine would prevent significant animal losses, especially in free-ranging farming, a common management practice worldwide.

## Data availability statement

The original contributions presented in the study are included in the article/Supplementary material, further inquiries can be directed to the corresponding author/s.

## Author contributions

AD: Conceptualization, Data curation, Investigation, Methodology, Project administration, Writing – original draft, Writing – review & editing. AF-S: Data curation, Methodology, Writing – review & editing. GA: Investigation, Methodology, Project administration, Writing – review & editing. AC-C: Methodology, Supervision, Validation, Writing – review & editing. GF: Conceptualization, Resources, Supervision, Validation, Writing – review & editing.
