# Supplementary material for: Tick paralysis induced by Ixodes gibbosus: enigmatic cases in domestic mammals from Cyprus
Source: Front Vet Sci. 2024 Jun 6;11:1416501. doi: 10.3389/fvets.2024.1416501 (PMC11189028; doi:10.3389/fvets.2024.1416501)
Supplement: Supplementary file 1 [file Data_Sheet_1.PDF]

ARISTOTLE UNIVERSITY OF THESSALONIKI

Anastasia Diakou, DVM, PhD
Professor
Laboratory of Parasitology and Parasitic Diseases
School of Veterinary Medicine, Faculty of Health Sciences
Aristotle University of Thessaloniki
Thessaloniki, 54124 Greece
Tel.: +302310 999927, *e-mail*: diakou@vet.auth.gr

Thank you for participating in information collection regarding our investigation about tick-paralysis. For the questions below, you can choose more than one answers.

Q1. Do you own or take care of farm animals?
☐ Yes ☐ No

Q2. If the answer in Q1 was "yes", what kind of animals?
☐ Sheep ☐ Goats ☐ Dogs
☐ Cats ☐ Other (define): ___________

Q3. In which area do these animals live?
……………………………………………………………

Q4. Are you aware of tick-paralysis?
☐ Yes ☐ No

Q5. Have you, personally, seen a case of tick-paralysis?
☐ Yes ☐ No

Q6. Have you heard other referring to tick-paralysis cases?
☐ Yes ☐ No

Q7. In what kind of animals have you seen tick-paralysis?
☐ Sheep ☐ Goats ☐ Dogs
☐ Cats ☐ Other (define): ___________

Q8. In what kind of animals have you heard of tick-paralysis?
☐ Sheep ☐ Goats ☐ Dogs
☐ Cats ☐ Other (define): ___________

Q9. If the answer in Q5 was "yes", what made you believe that a paralysis case was caused by ticks?
☐ Sudden onset of paralysis ☐ The animal had no medical history ☐ the fact that tick-paralysis occurs in my region ☐ the animal recovered soon after tick removal

Q10. How often to you observe tick-paralysis?
☐ Just once ☐ Rarely ☐ Approx. yearly ☐ Every 2 years ☐ Every 3 years ☐ Every 4 years ☐ Every 5 years ☐ Every 6 years ☐ Every 7 years ☐ In a period longer than 7 years

Q11. What season of the year have you seen tick-paralysis case(s)?
☐ Autumn ☐ Winter ☐ Spring
☐ Summer

Q12. Have you experienced animal death(s) due to tick-paralysis?
☐ Yes ☐ No

Q13. If the answer in Q12 was "yes", what kind of animals have you seen dying due to tick-paralysis?
☐ Sheep ☐ Goats ☐ Dogs
☐ Cats ☐ Other (define): ___________

Q14. If the answer in Q12 was "yes", what is the number of animals that suffer or/and die due to tick-paralysis each occurrence period?
☐ 1-2 ☐ 2-5 ☐ 5-10 ☐ > 10 (define approx.: _____):

Q15. I case you encounter tick-paralysis in your animals, what are your actions?
☐ No action-the animals recover spontaneously
☐ Nothing can help the animals, death is inevitable
☐ Tick removal ☐ Acaricidal treatment ☐ Other (define): ___________________________

Q16. Did you seek veterinary help for tick-paralysis case(s)?
☐ Yes ☐ No

Q17. Do you take preventive measures for tick-paralysis?
☐ No ☐ Regular acaricidal treatment ☐ Occasional acaricidal treatment ☐ acaricidal treatment when tick-paralysis occurs ☐ Other (define): ________________

Q18. If you own a small ruminants farm, of what type is it?
☐ free-ranging ☐ semi-intensive

Thank you for your time!
